# 

**Published:** 1831

**Authors:** 


### INDEX.

A.

Acetate of Lead in Ulcerated Phthisis, 444.

Acetate of Lead relieving Pulmonary Disease. By Dr. Bush, 485.

Absorption of the fluids, Life supported by, 113.

Abnormal Menstruation, 106.

Alteration of the Blood in Yellow Fever, 417.

Acid, Oxalic, Lozenges, 434.

Ammonia, conefficacy of, as an antidote to Prussic Acid, 263.

Amaurosis, 418, 428.

Aneurism, Carotid; Anticardiac operation, 138.

Apomea Jalap, the product of, 124.

Animal Putrefaction, Influence of Electricity on, 442.

Armstrong, Dr. John, Life and character of, 60.

Artificial Eyes, 419.

Aromatic Syrup of Rhubarb, Tincture of do., 432.

Arteries, unusual distribution of, 157.

Apthous Sore Mouth, by Dr. Cook, 346.

Aphonia Intermittent, 114.

—of thirty years duration 115.

Atlee's Case of Tracheotomy, 23.

of Obstetrics, 154.

B.

Bancal on Lithotrity, 496.

Bard on Chronic Dysentery, 317.

Bush's Case of Ruptured Bladder, 194.

—Pthisis cured by Lead, 489.

Begin's Therapeutics, 518.

Bark adulterated with Iron, 431.

Bennet, Dr. J. C., on Apthous Sore Mouth, 346.

Belladonna proving a Poison, when used as an Injection, 430.

Bladder, Rupture of, terminating fatally, 194.

Bilious Fever, alternating with Gonorrhea, 420.

— Finley on the Pathology of, 39.

Modified by Hysteria, 492.

Biographical Notice of Loemering, 4[illegible]5.

Armstrong, 60.

Wells, 4[illegible]8.

Godman, 596.

West, 596.

Parey, 313.

Brent on the Removal of a tumor, 487.

Broussais, Strictures on. By Dr. Eberle, [illegible]

Blood, Altered in Yellow Fever, 417.

Bone, Regeneration of, 112.

Lodged 48 days in the Trachea, 429.

New Mode of Cleansing, 589.

C
Catalepsy, 572.
Cataract, Alternating with Diabetes, 132.
Cæsarian Operation. 277.
State of the Uterus 3 years after, 106.
Carotid Aneurism, 138.
Charbon, Description of, 35.
Carmichael on Venereal Diseases, 545.
Clavicle Dislocated forwards, 122
Croup in the Adult, 1[illegible]1.
Tracheotomy in, 23.
Chloride of Magnesia, 288.
Chorea, 435.
Combustion, Spontaneous, 283.
Conical Cornea. By Dr. Staughton, 188.
Copaiva, Purity of, 444.
Cornea, Opacities of, 592.
Cancer of the Rectum, 586.
Carious Rib, Excision of, 582.
McClellan's Case of, 479.
Cytocele, 281.
D
Dewees's Practice of Physic, 348.
Delirium Tremens, 140.
Traumatic, 453.
Diabetes Alternating with Cataract, 124.
Dislocation of the Clavicle, forwards, 122.
Drake's Cases of Burn, 48.
Exophthalmos, 197.
Life of Godman, 596,
Dry Gangrene, 28—156.
Dupuytren's Mode of Removing Polypi of the Uterus, 11[illegible].
Treatment of Hernia Humoralis, 137.
Dysentery, Bard's Case of, 431.
Dysmenorrhea, Eberle on, 161.
Pregnancy occurring during, 584.
Duffin on the Physical Education of Females, 404.
E
Economic Lighting, 596.
Eberle's Observations on Fever, 9.
On Rhus Toxicodendron in Paralysis, 33.
On Dysmenorrhea, 161.
Ectropium, New operation for, 5[illegible]2
Ellison on a Disease called Charbon, 3[illegible].
Epilepsy, Staughton's Case of, 340.
Epidemic Pleurisy. By Dr. Kimball, 473.
Excision of the Elbow Joint, 138.
Of the knee Joint, 287.
Of the Sternum and Carious Ribs. By Dr. McClellan, 479.
Of Upper Jaw, 438.
Of a Carious Rib, 582.
Of the Nerves in Neuralgia, 275
Exopthalmos. By Dr. Drake, 197.
Extirpation of the Inverted Uterus, 152.
Extirpation of the Parotid, 405.
Of Cancer of the Rectum, 580.
Eyes, Artificial, 449.
Humors of, evacuated by a wound, without loss of sight, 435.
Treatment of certain injuries of, 426
F
Formulæ, 433
Femur, Fracture of, treated by Ligature, 588
G
Gerhard on Endermic Administion of Medicine, 289
Gonorrhea alternating with Bilious Fever, 426
H
Haldeman's Case of Fracture of, the Occipital Bone, 328
Hematosis, Jackson's Observations on, 144
Hernia of the Stomach, 423
Humoralis, 437
Of the Bladder, 281
Dgianosis of, 273

Hydrocyanic Acid, Inefficacy of Ammonia as an antidote for, 263
Hydrocephalus, Tapping in, 440.
Hysteria, Modification of Fever Produced by, 492

I

Infanticide, 134
Iodine, Rives on. 176
Morbid Effects of, 424
Iodide of Mercury, Lupus treated by, 280
Inflammation, Gastric and Intestinal, Diagnosis of, 279
Iris, Congenital, Absence of the 111

J

Jalap, the Production of an Opomea, 124

K

Kimball on Bilious Pleurisy, 473.
Case of Poisoning from the local application of Cor. Sub. 483.

L

Lead, Mode in which the poison of, is introduced into the system, 450.
Acetate of, in Ulcerated Phthisis, 444.
Symptoms of Phthisis reliev- by Acetate of, 485.
Life, supported for a length of time by Absorption, 113.
Lindsey's Case of Dry Gangrene, 28, 156.
Litter's Case of Nervous Irritation, from the Sting of a Yellow Jacket, 192.
Lithotrity Dr. Smith's Observations on, 462.
Bancal on, 496.
Lupus, treated by Iodide of Mercury, 280.
Lymphatics communicate with the Veins, 106.

M

Magnesium, Preparation of the Chloride of, 288.
Male, Influence of extending beyond a single Impregnation, 115.
Mania, Puerperal, 286.
Military Surgery, Hennen's, 376.
McCulloch on the Diseases of Malaria, 213.
Menstruation, Abnormal, 106.
Finley on, 332.
Monomania, Homicidal, 576.
Report of the Trial of Jonathan Martin, 296.

N

Nasal Canal, Lower Orifice of, 289
Nævus, cured by Seton, 592.
Wardrop's Mode of treating, 435.
Nerves, Excision of, in Neuralgia, 275.
Division of the Maxillary, 456.
Nicotin, the active principle of Tobacco, 123.
Nervous Affections, Cold Affusions in, 578.

O

Obstetrics, Atlee's Case of, 154.
Occipital Bone, Fracture of, 328.
Opacities of the Cornea, 592.

P

Parotid Gland, Extirpation of, 465.
Parey, Life of, 313.
Pathology of Dysmenorrhea, 161.
Of Fever, 9.
Phlegmasia Dolens in a Male, 424.
Penis, removal of by Ligature, 589.
Poisoning by Belladonna used as an Injection, 438.
Evidence of, 265.
By Mouldy Bread, 596.
Pregnancy, distinctive marks of, 476.
Occurring during Dysmenorrhea, 594.
Puberty, Period of in Women.
Puerperal Mania, Treatment of 286.
Pulmonary Disease relieved by Lead, 489.

R.
Rectum, Excision of, 580.
Reunion of divided parts,
Remarkable case of,
Rupture of the Bladder, 104.
Of the Uterus, recovery from, 416.
Rhubarb, test of the different kinds of, 591.
Aromatic Tincture of, 432.
Aromatic Syrup of, 437.
Rutgers Medical college, 300.
S.
Salicia in the treatment of Intermittent Fever, 285.
Not an alkali, 584.
Salivation promoted by Acids, 343.
Sight recovered after evacuation of the humors of the eye, 435.
Smith on Lithotrity, 461.
Soemmering, Life of, 455.
Spermatic chord, strangulation of, 585.
Spasmodic Asthma relieved by Inhalations, 596.
Spontaneous combustion, 28.
Staughton's case of Epilepsy, 340.
Conical Cornea, 188.
Life of Parey, 313.
Strabismus, 429.
Stomach, recovery from a wound of, 441.
Strychnine in Amaurosis, 418.
In Chronic Diarrhea, 131.
Submaxillary Nerves, division of, 455.
Sulp. Quina, Intoxicating effects of, 446.
Syphilis, Hennen's observations on, 545.
T.
Tapping in Hydrocephalus, 284.
Taste, seat of, 444.
Tetanus caused by a foreign body lodged in the Ulnar Nerve, 437.
Therapeutics, Begin's, 518.
Tracheotomy in croup, 23.
Transfusion, 443.
Traumatic Delirium, 453.
V.
Vaccination, Finley on, 166.
Vena Cava, Inflammation of, 580.
Veins, M. Berard on, 571.
Vespa Crabro, Irritation produced by the sting of, 192
Venesection in the cold stage of Intermittent Fever, 425.
Venereal Diseases Carmichael on, 545.
Urachus,
Uterus, Recovery from Rupture of, 416.
Extirpation of, 119.
Excision of the Inverted, 152.
Dupuytren's mode of removing Polypi of, 117.
Utero Gestation, protracted case of, 420
Wardrop's mode of treating Nævus, 435.
Wells, Dr. John, Life of, 458.
Wedemeyer on the circulation, 107.

